# Kickoff to Conflict: A Sequence Analysis of Intra-State Conflict-Preceding Event Structures

**DOI:** 10.1371/journal.pone.0122472

**Published:** 2015-05-07

**Authors:** Vito D'Orazio, James E. Yonamine

**Affiliations:** 1 Institute for Quantitative Social Science, Harvard University, Cambridge, MA, USA; 2 RPX Corporation, San Francisco, CA, USA; East China University of Science and Technology, CHINA

## Abstract

While many studies have suggested or assumed that the periods preceding the onset of intra-state conflict are similar across time and space, few have empirically tested this proposition. Using the Integrated Crisis Early Warning System's domestic event data in Asia from 1998–2010, we subject this proposition to empirical analysis. We code the similarity of government-rebel interactions in sequences preceding the onset of intra-state conflict to those preceding further periods of peace using three different metrics: Euclidean, Levenshtein, and mutual information. These scores are then used as predictors in a bivariate logistic regression to forecast whether we are likely to observe conflict in neither, one, or both of the states. We find that our model accurately classifies cases where both sequences precede peace, but struggles to distinguish between cases in which one sequence escalates to conflict and where both sequences escalate to conflict. These findings empirically suggest that generalizable patterns exist between event sequences that precede peace.

## Introduction

In late 2010 and early 2011, the “Arab Spring” was sweeping across the Middle East and North Africa. By March 2011, popular uprisings had led to the removal of dictatorial regimes in Tunisia and Egypt, and attention turned to Saudi Arabia and the planned “Day of Rage” set to occur March 11. In the days prior, great emphasis was placed on forecasting future political instability in Saudi Arabia due to its global economic and political influence. In many ways, the events in Saudi Arabia resembled those in nearby countries like Egypt, Libya, and Tunisia: young, progressive citizens leveraging technology to orchestrate demonstrations aimed at ousting the dictatorial regime by any means necessary. However, certain aspects of Saudi Arabia—such as its extreme oil wealth and small population—differentiate it from most other states.

Given these differences, an analyst attempting to forecast the degree of political instability is faced with a difficult question: to what extent should one look for similarities between patterns of events in Libya and Tunisia in order to build forecasts for Saudi Arabia? Indeed, this type of question exists every time an analyst attempts to forecast political instability, and speaks to a broader and arguably more important question for both the policy and academic community alike: to what extent do similarities exist between sequences of events that precede the onset of intra-state conflict in various states over time?

Although the ramifications are relevant to both qualitative and quantitative conflict forecasting approaches, it is also theoretically important to our understanding of conflict processes. If patterns do exist between sequences of events, it suggests that similar causal processes may exist across highly diverse societies over time. However, if discernible similarities do not exist, it may support some qualitative arguments that conflicts are unique and should be studied (or forecasted) on a case-by-case basis.

Despite well-developed quantitative literature on intra-state conflict, including both state-year, large-N analyses and sub-state, sub-annual event data approaches, relatively little attention has been has been given to address the extent to which similarities exist between sequences of events that precede conflict occurring across a broad spectrum of countries. The central goal of this paper is to provide an objective, methodologically rigorous approach to answer this question. To do so, we leverage the strength of large-N and event data studies by building and then comparing nuanced event sequences—some of which precede an onset of intra-state conflict while most lead to intra-state peace—for a range of diverse countries to determine the extent to which patterns exist among the sequences.

A number of earlier studies have provided a theoretical foundation for the use of event sequences as well as empirically demonstrated that sequences can help explain and forecast various political outcomes-of-interest. For example, [1] and [2] discuss the relevancy of analyzing events in sequence. [3–6] utilize variations of sequence approaches. However, none of the extant sequence analysis studies focus specifically on events preceding intra-state conflict onset or utilize methodologies designed to test for the existence of patterns within high-dimensional sequences occurring across a diverse set of states. In this respect, our application of the sequence analysis is unique from others.

A critical aspect to carrying out our approach is data availability, since it requires nuanced event data for a large number of countries over a sufficiently long period of time. In 2011, the Defense Advanced Research Project Agency (DARPA) funded Integrated Crisis Early Warning System (ICEWS) project released a data set containing over 2 million, daily level events in a who-did what-to whom, when format for the 29 Asian states under the area of responsibility for the Pacific Command division of the United States military from 1998 through 2010. These data allow us to build and compare highly nuanced event sequences occurring across a large number of diverse states for an extensive period of time.

To convert these raw event data into meaningful sequences, we aggregate events into eight weekly and monthly level sequences that reflect the number of politically relevant events occurring between the government and opposition groups in each state. Next, using an application of sequence analysis, we measure the similarity between pairs of sequences using three metrics: Euclidean, Levenshtein, and mutual information. Finally, we use the scores (which reflect the degree of familiarity between sequences) as predictors in a bivariate logistic regression to estimate the likelihood that neither, one, or both sequence in each pair precede an onset of intra-state conflict in the following month.

We find that our model accurately classifies cases where both sequences precede peace, but struggles to distinguish between cases in which one sequence escalates to conflict and where both sequences escalate to conflict. These findings suggest generalizable patterns of non-escalatory events exist across time and space. Furthermore, the hypothesis that states tend to follow unique escalations towards conflict cannot be rejected, suggesting generalizable patterns of escalatory event sequences do not exist across time and space. Rather, states may follow unique escalations towards conflict. Additionally, we find that some states experience intra-state conflict without warning. That is, some states see no changes in their event structures preceding a conflict onset. These “masked” conflicts are not possible to predict using the ICEWS event data, and may suggest a class of conflict that is essentially unpredictable with current research tools.

## Methods

### Motivation

Since the late 1990s, a wealth of large-N quantitative studies have emerged with the primary goal of finding statistically significant relationships that hold across a large number of states. [7] and [8] offer two canonical efforts to analyzing intra-state conflict onset that utilize this framework and demonstrate the strengths and limitations of this approach. In both studies, the authors utilize time-series cross-sectional (TSCS) datasets with independent variables that primarily vary cross-sectionally between states, rather than temporally within the same state, and a binary dependent variable reflecting intra-state conflict onset. The primary strength of this type of large-N analysis is that it has revealed that certain structural variables such as rough terrain, GDP per capita, and population size have statistically significant and relatively stable effects on the likelihood of intra-state conflict onset across time and space ([8] and [7] cover 161 states from 1946–1992 and 123 states from 1965–1999, respectively). For example, all else being equal, poorer states with mountainous terrain and large populations are more likely to experience onsets of intra-state conflict.

Although these approaches have contributed greatly to our understanding of intra-state conflict, their main weakness is that they do not account for the actual, sub-annual level events that tend to lead to conflict. For example, population and rough terrain may affect the likelihood of conflict onset in the aggregate, but these factors by themselves do not *cause* onsets of conflict in the sense that they are present both before and after conflict occurs. Rather, it is specific events, such as protests, demonstrations, broken peace talks, etc., that actually triggers conflict.

Conversely, event data approaches tend to focus on highly specific, sub-annual (often weekly or monthly) level variation in a broad spectrum of politically relevant events, ranging from verbal threats to diplomatic meetings to violence. However, these studies tend to focus on events occurring in a single state or multiple states involved in a joint conflict. For example, [9] and [10] analyze the Arab-Israeli conflict, [11] and [12] focus on Serbia, and [13] is interested in Chechnya. In [10], data from more than one state are interpreted jointly, whereas in other studies, such as [4], multiple states are modeled individually and then empirical findings are informally compared. The primarily goal of most of these studies is to build accurate, objective, and temporally nuanced forecast of conflicts.

We build on previous efforts by using fine-grain, comprehensive event data at the sub-state level to compare sub-annual level event sequences across a diverse set of states. Our approach is an attempt to move towards merging the predominantly cross-sectional, large-N approaches with the time-variant, event data approaches.

### Event Data

The event data used in this research come from the DARPA-funded Integrated Conflict Early Warning System [14, 15]. ICEWS has invested substantial resources in all areas of event data development including the development of the automated coders, actor and action dictionaries, and for access to a very large collection of news documents. Its investment into event data development put ICEWS on a scale not seen in prior event data coding efforts. As [14] notes,

…the ICEWS performers used input data from a variety of sources. Notably, they collected 6.5 million news stories about countries in the Pacific Command (PACOM) AOR [area of responsibility] for the period 1998–2006. This resulted in a dataset about two orders of magnitude greater than any other with which we are aware. These stories comprise 253 million lines of text and came from over 75 international sources (AP, UPI, and BBC Monitor) as well as regional sources (*India Today, Jakarta Post, Pakistan Newswire*, and *Saigon Times*).

The range was eventually extended through 2010, where this study concludes, and beyond. The initial phase of ICEWS used both the Tabari and the VRA automated coding technologies, with the latter used as one of the sources to generate the ICEWS ‘events of interest’ [16–20]. These ‘events of interest’ comprise the dependent variable in this study and are discussed in more detail below. In the second phase of the project, Lockheed-Martin developed a direct translation (with some bug corrections) of Tabari into Java called Jabari, and the data produced by that system are the data utilized here [21]. These data are coded according to a 15,000-item action dictionary using the CAMEO typology and an 8,000-item actor dictionary to code for a broad range of actors, including but not limited to military, police, rebel groups, and civilians [22]. As a result of the inclusion of local and regional news sources as well as the comprehensive actor dictionary, the ICEWS dataset provides sufficient sub-state actor nuance and spatial coverage for us to address our central research question.

### Aggregation Techniques

Every event coded by the ICEWS project is coded in a “who did what to whom and when” format. This raw event data provides researchers with a tremendous amount of flexibility regarding aggregation techniques for making the data usable for empirical analysis and exists across three areas of aggregation: actors, actions, and time. In this section, we provide a brief overview of common aggregation approaches in the literature and then explain the choices we make regarding actors, actions, and time to build our event sequences.

#### Actor Aggregation

Raw event data provide information regarding the actors involved with the action. Typically, this is formatted in terms of a ‘source’ and a ‘target,’ although some actions are non-directional. Given this, researchers must determine the actors of interest between whom an event must occur in order for that event to be included in their study’s empirical models. At a minimum, this means events must involve at least one actor affiliated with a country of interest. The justification for this minimal level of actor aggregation is clear: a study focusing on Israeli-Palestinian conflicts would not want to include events between Aceh rebels and the Indonesian army, as these are not relevant to the conflict of interest. Although excluding Indonesian rebel activity is obvious, more difficult decisions exist, such as whether or not to include events between members of the Lebanese and Syrian armies or between the governments of the United States and Iran. Other questions exist regarding which domestic actors are relevant to the study of intra-state conflict. In the example above, we would want to exclude a bar fight between two Israeli citizens over the outcome of a soccer match but not a fight occurring between an Israeli soldier and a Palestinian government official.

Unfortunately, the majority of studies utilizing event data provide incomplete explanations regarding their actor aggregation. Others provide a conceptual discussion of actors of interest but do not include how these concepts are operationalized. For example, [23] discusses “mass” and “state” actors; [24] focuses on “governing elites,” “mass followship,” “disadvantaged groups,” etc.; [25] addresses “kindred groups,” “communal groups,” etc; [4] discusses “government” and “dissidents”. Given the lack of formal precedence regarding transparency in actor aggregation techniques, we attempt to explain our approach as clearly as possible.

Every coded event in the ICEWS dataset contains two actors: a source and a target. Because ICEWS uses the CAMEO coding ontology, each actor is coded using a three-tiered scheme. The first tier is provided for all actors and reflects national identity, which we require to be identical for both the source and the target. This ensures that we only analyze events occurring domestically. Additionally, the ICEWS dataset often includes a second tier of information with sub-national level descriptions and, occasionally, a third tier code that is usually related to the identity of the individual actor. We drop the third tier and select only relevant groups of actors from the second tier. Specifically, we build three main “classes” of actors based on their second tier categories for the events that occur domestically:

- *Government*, which includes actors identified by ICEWS as:
  - [*MIL*]—Military
  - [*POL*]—Police
  - [*GOV*]—Member of government with no additional information provided
  - [— — —]—Actors who do not receive secondary or tertiary codes
- *Rebels*, which includes actors identified by ICEWS as:
  - [*INS*]—Insurgents
  - [*SEP*]—Separatists
  - [*REB*]—Rebels
- *Other*, which includes all other domestic actors including but not limited to:
  - [*CIV*]—Civilians unaffiliated with another group
  - [*BUS*]—Individuals identified as a business person
  - [*EDU*]—Students and teachers

The inclusion of [— — —] into government actors is consistent with CAMEO’s ontological assumptions [22]. The domestic events occurring between the government and rebel groups and between rebel groups and other non-governmental actors are the primary interactions comprising escalatory processes in intra-state conflict. Therefore, these are the actors used. They comprise two classes of actor aggregations: GOV-REB and REB-OTH.

#### Action Aggregation

Event data studies either scale actions, assigning them a score on a conflict-cooperation continuum, generate event counts reflecting the number of events that occur within conceptually unique categories, or utilize a mixture of the two techniques, as in [23, 26]. The Goldstein Scale, which is the most commonly used scaling technique within the event data literature, assigns a value to all events coded under the WEIS scheme on a -10 to +10 conflict-cooperation continuum [27]. On this scale, a -10 reflects the most conflictual event and +10 indicates the most cooperative event. Despite its widespread use [11, 13, 17, 27, 28], the Goldstein Scale requires additional levels of aggregation beyond the initial scaling, which leads to a number of operational difficulties. For example, consider a day during which an armed killing (which receives a -10 score) and a peace-treaty signing (which receives a +10 score) occur between the same actors. The sum and the mean of the Goldstein scores—two of the most common approaches for working with scaled data—would result in coding of 0, the same score that days with no activity receive. Theoretically, it is apparent that the nature of events occurring on a day comprised of purely neutral events and a day with a -10 event and a +10 event are fundamentally different.

While this example of two events perfectly canceling is hypothetical, the problem of violent events masking the concurrent presence of cooperative actions—notably negotiations occurring during periods of ongoing violence—is very real and occurs frequently. This is further complicated by the fact that some actions like “comments” and “meetings” have Goldstein scores that are small in magnitude, whereas violent events have a scale score of -10. Consequently a small amount of violence can mask a lot of talking.

Because of these problems with scaled data, a number of other studies utilize count structures [9, 29, 30]. [31] put forth the first event data count structure by placing all events into one of the four conceptually unique, mutually exclusive categories. The original coding was done using WEIS actions, which we translate to the CAMEO categories listed below:

- *Verbal Cooperation*: The occurrence of dialogue-based meetings (e.g. negotiations, peace talks), statements that express a desire to cooperate or appeal for assistance (other than material aid) from other actors. CAMEO categories 01 to 05.
- *Material Cooperation*: Physical acts of collaboration or assistance, including receiving or sending aid, reducing bans and sentencing, etc. CAMEO categories 06 to 09.
- *Verbal Conflict*: A spoken criticism, threat, or accusation, often related to past or future potential acts of material conflict. CAMEO categories 10 to 14.
- *Material Conflict*: Physical acts of a conflictual nature, including armed attacks, destruction of property, assassination, etc. CAMEO categories 15 to 20.

Overall, these event count structure makes fewer assumptions than the Goldstein Scale about the impact of events. Moreover, because negative counts are impossible, this approach does not suffer from problems of generating sum or mean scores that affect the Goldstein Scale. Although this count approach is simpler than scaling methods, [9] and [12] still find strong empirical results with data aggregated into these four categories. Therefore, an event count technique parallel to [31] but adjusted for the CAMEO categories as indicated above is employed. This approach provides a straightforward representation of the number and type of events that occur while making the fewest assumptions about future effects of the events.

#### Temporal Aggregation

Researchers must temporally aggregate data in order to perform empirical analyses at levels appropriate for both their theoretical and empirical models. All of the previously mentioned event datasets code the exact day on which events occur. As the specific time-of-day that events occur is not reported, events must at the very minimum be aggregated to the daily level [9, 11, 12]. Weekly [32, 33], monthly [28, 34], quarterly [26], and even annual level aggregations are also found in the literature.

Often, the level of temporal aggregation is more subjective than the other areas of aggregation. Furthermore, a number of studies have shown that different temporal aggregations can affect the empirical results [35–37]. Therefore, it is important to both justify the level of aggregation used and, if possible, use multiple levels as robustness checks. We use both weekly and monthly levels of temporal aggregation. Doing so allows us to capture event fluctuations that occur across small periods of time and is more robust to noise than daily level aggregations. Furthermore, it allows for robustness checks across two levels of aggregation. [4] and others have attempted to move beyond traditional, calendar-based practices by aggregating according to empirically discernible temporal clusters within the data. While we do not attempt such an aggregation here, it presents an interesting alternative.

To generate weekly and monthly-level data, we sum the event counts for all relevant actor combinations (GOV-REB, REB-OTH) across each action (verbal cooperation, material cooperation, verbal conflict, material conflict), which results in eight sequences of event counts for each unit-of-analysis (the period preceding each conflict onset and the period preceding selected peaces). For example, Table 1 contains sequences that precede a conflict onset in Indonesia. This represents the data after sequence *construction* but prior to sequence *analysis*.

**Table 1:** Event Count Structures Preceding Conflict in Indonesia.

		Week 45, 2001—Week 5, 2002												
	**Actor-Action**	**45**	**46**	**47**	**48**	**49**	**50**	**51**	**52**	**1**	**2**	**3**	**4**	**5**
GOV-REB	Verbal Cooperation	0	0	5	0	1	0	0	0	1	0	1	4	4
	Material Cooperation	0	0	0	0	0	0	0	0	0	0	1	1	0
	Verbal Conflict	0	0	0	0	0	0	0	0	0	0	0	2	0
	Material Conflict	4	1	0	0	0	0	0	0	0	4	1	3	4
REB-OTH	Verbal Cooperation	0	0	0	0	1	0	0	0	0	0	0	3	0
	Material Cooperation	0	0	0	0	0	0	0	0	0	0	0	0	0
	Verbal Conflict	0	0	0	0	0	0	0	0	0	1	0	0	0
	Material Conflict	0	0	0	0	1	0	0	0	0	0	2	0	0

Our approach to sequence construction differs from those utilized in the related sequence analysis literature in two main ways. First, our approach is unique in that it measures the number of events that occur within fixed units of time (weeks and months), whereas other studies such as [3], [1], and [4] focus on the sequential ordering of events regardless of time, which means that they are unable to explicitly account for the amount of time that transpire between events. Second, our approach allows for multiple events to occur between multiple actors simultaneously, whereas the extant studies place events in a sequential order. Third, we build sequences from event counts as opposed to a single event [1, 3, 38] or a conflict-cooperation scaled value [4]. We argue that utilizing counts captures the greater variation in the intensity of actions. Overall, we believe our approach to sequence construction is a more effective way capture the complexity of event structures that precede intra-state conflict onsets.

## Results

### Research Design

Our research design is a combination of a sequence analysis and regression approach. Using a sequence analysis, we construct variables that reflect a degree of similarity between pairs of sequences where both precede peace, one precedes peace and one precedes conflict, and where both precede conflict. After comparing the scores for each pair type, we test for the ability of event structures to distinguish pair types using a bivariate logistic regression.

The conflict onset variable (GTDS) is a binary measure constructed from a ground truth measure of intra-state conflict that has been developed by DARPA. Table 2 is a complete listing of all conflict onsets in our data. The objective of ground truth data in conflict studies is to provide a more immediate representation of a conflict than can be provided purely by journalistic or historical reporting. Although DARPA has chosen not to publicly release the explicit coding rules for the GTDS, it has been used as a dependent variables in a number of conflicted forecasting publications, some of which are specifically designed for policy planning (see [14, 39–41] for other uses of the GTDS). The GTDS contains four categories of intra-state conflict at the monthly level: Rebellion, Insurgency, Domestic Crisis, and Ethno-religious Violence.

**Table 2:** GTDS intra-state conflict Onsets.

**State**	**Onset Date**	**State**	**Onset Date**
Bangladesh	May, 1998	Bangladesh	January, 2004
Bangladesh	January, 2008	China	January, 2003
Fiji	May, 2000	Fiji	January, 2001
Indonesia	November, 1998	Indonesia	January, 1998
Indonesia	February, 2001	Indonesia	August, 2005
Indonesia	November, 2006	Indonesia	January, 2006
Indonesia	March, 2006	Indonesia	August, 2008
Indonesia	April, 2009	Indonesia	June, 2009
Indonesia	March, 2009	India	January, 2001
India	January, 2003	India	January, 2004
India	July, 2007	India	February, 2008
Cambodia	September, 2001	Cambodia	January, 2001
South Korea	August, 2007	Laos	January, 2002
Sri Lanka	January, 2002	Madagascar	January, 2000
Madagascar	January, 2003	Madagascar	January, 2008
Madagascar	November, 2010	Myanmar	August, 2007
Myanmar	June, 2009	Myanmar	January, 2009
Myanmar	October, 2010	Myanmar	November, 2010
Nepal	November, 2005	Nepal	February, 2006
Nepal	June, 2010	Nepal	August, 2010
Philippines	February, 1998	Philippines	January, 2000
Philippines	January, 2005	Solomon Islands	April, 2000
Solomon Islands	January, 2002	Solomon Islands	April, 2006
Thailand	January, 2003	Thailand	January, 2005
Thailand	July, 2008	Thailand	September, 2008
Thailand	January, 2008	Thailand	November, 2009
Thailand	September, 2010		

For a state-month to be coded as an onset in our data, a positive observation in a single category must be preceded by at least three months of peace in that category. According to this operationalization of onset, it is possible for a state to experience two or more onsets in a given month so long as the onsets occur in different categories.

To establish pairs for comparison, we select all 13-week sequences in our dataset that precede conflict and a random sample of sequences that precede peace at a ratio of about 2:1 peace-preceding:conflict-preceding. For robustness, we have also constructed these sequences at the monthly level based on three-month periods. The 2:1 ratio is ad hoc and could bias the results towards increasing the number of predicted onsets relative to what would be predicted given a true random sample. However, the peace-preceding sequences must be sampled in some fashion to avoid excessive overlapping sequences, and given the small number of onsets generally analyzed in this literature we deem 2:1 an appropriate ratio. To avoid comparing sequences where there is very little to no rebel interactions (e.g., Australia), we only randomly sample from the 15 states that experience at least one onset. This results in 113 sequences that precede peace and 53 sequences that precede conflict onset. We then calculate the similarity scores for each pair of sequences, resulting in (1662), or 13,695 pairs (observations), each with eight explanatory variables (the distances between the eight count variables of each sequence).

The Euclidean distance, the most intuitive of the three and the one through which the primary results of this paper are reported, is seen in Eq (1).

$$\begin{array}{c} \sqrt{\sum_{i=1}^{13}{\left(A_{ij}-B_{ij}\right)}^{2}} \end{array}$$ (1)

To illustrate this process, consider the example in Table 3. For State A, we have eight actions measured over 13 weeks. For each of these eight actions, we calculate the Euclidean distance with the corresponding action in State B. The Euclidean distance is a conceptually and mathematically straightforward approach used to calculate the distance between two vectors, and has been used in the study of International Relations in other applications [42]. This robust approach is applied to vectors according to the formula in Eq (1), in which *A* and *B* are the two states in the pair, *i* is the week/month, and *j* is the action variable (e.g., verbal cooperation). As can be seen in Table 3 for a single action, the Euclidean distance would be calculated for the sequences {5,2,3,5,2,3,0,2,1,1,2,0,0} and {4,0,1,2,1,0,0,0,1,0,0,0,4}. In this example, the Euclidean distance approximately equals 7.28.

**Table 3:** Vectors of Verbal Cooperation Counts for State A and State B.

**Actor-Action**	**Weekly Counts**												
GOV-REB (State A)													
Verbal Cooperation	5	2	3	5	2	3	0	2	1	1	2	0	0
GOV-REB (State B)													
Verbal Cooperation	4	0	1	2	1	0	0	0	1	0	0	0	4

Despite its generality, the Euclidean distance may not adequately capture similarity given the underlying event sequences are occurring in different places and at different times. For example, the distance between states with high levels of journalistic reporting and states with low levels will always be large, even if the underlying event structures are comparable. Or, events in one country may unfold over a period of months while events in another country, although similar in structure, unfold over just a few weeks. Additionally, as a second order metric the Euclidean distance may not capture higher order interdependencies in the event structures. The sequence analysis literature across disciplines is rich in applications of distance and related metrics to account for higher order interdependencies [43–45]. To further explore such interdependencies and to extract the most information from our data, we also use the Levenshtein distance and mutual information to compare event sequences.

The Levenshtein distance is an edit distance, developed and used primarily in computer science [46, 47] but with applications to sequence analysis as well [48, 49]. The intuition behind the general class of edit distances is to measure the number of computer calculations necessary to transform one string, or vector, into another. The available transformations are *shifts* and *substitutions*. A shift is an insertion that effectively shifts the sequence to the left or right. So, if two sequences are identical except for the fact that one sequence began at time *t* and the other at *t*+1, the Levenshtein distance algorithm would simply shift the sequence in the appropriate direction, just a single calculation. Substitutions are also measured as a single calculation, regardless of the “distance” between the numbers (or characters) being substituted. That is, if two sequences are perfectly aligned with the exception of the *n* ^*th*^ character, the Levenshtein distance algorithm would simply substitute the *n* ^*th*^ character, again just a single calculation. In other words, whereas the Euclidean distance takes into account how far apart the *n* ^*th*^ characters in each sequence are, the Levenshtein distance views this as a single calculation regardless of the physical number.

Mutual information is an entropy-based metric from Information Theory [50]. Rather than measuring a geometric or edit distance, mutual information measures the reduction in uncertainty (entropy) about Sequence A that may be ascertained from knowledge of Sequence B. This robust metric may be particularly useful in instances where both the Euclidean and Levenshtein distances are large, but high order interdependencies still exist that the metrics do not capture. In such cases, mutual information may discover a relationship that may be useful in characterizing the sequence outcomes as something different than what the Euclidean or Levenshtein may produce. Here, we apply Linfoot’s normalization of mutual information [51], as described in [43]. Specifically, mutual information is defined in Eq (2), where X and Y represent our discrete sequences of events and *I*_*N*_ is the normalization of the mutual information function.

$$\begin{array}{ccc} I(X,Y) & = & \sum_{x}\sum_{y}p\left(x,y\right)log\frac{p(x,y)}{p(x)p(y)}\\ I_{N}\left(X,Y\right) & = & \sqrt{1-e^{2I(X,Y)}} \end{array}$$ (2)

### Sequence Comparison

We calculate the similarity scores among all possible pairs of peace-preceding sequences and conflict-preceding sequences, which results in three types of sequence pairs for comparison:

- Type 0—Neither of the compared 13-week sequences precede conflict onset
- Type 1—One of the compared 13-week sequences precede onset and the other did not
- Type 2—Both of the 13-week sequences precede conflict

Using the Euclidean Distance to build intuition for the approach, imagine a case where event sequences in periods preceding conflict are similar across time and space. In such cases, Type 2 distances should be comparatively small. Conversely, large distances between Type 2 pairs suggests that event sequences of periods preceding conflict onset may not follow similar trends across time and space. The same logic applies to distances generated by Type 0 pairs, which will be small if periods proceeding peace exhibit similar event structures. Lastly, distances generated by Type 1 pairs reflects the extent to which periods preceding peace and conflict onset vary, meaning that small distances (relative to Type 0 and Type 2 distances) suggest that we may be unable to discern between sequences preceding peace and conflict. Thus, if sequences preceding peace are similar and sequences preceding conflict are similar (but different from those preceding peace), an empirical model will struggle to correctly differentiate between Type 0 and Type 2 pairs in an out-of-sample context. If we assume that sequences preceding peace are similar (an assumption with strong empirical support), then the extent to which patterns exist between sequences preceding a conflict onset depends on the out-of-sample *misclassification* of Type 2 pairs, explained by the two conditions below.

- **Condition 1**: If patterns *exist* between sequences preceding conflict, the empirical model will struggle to differentiate between Type 2 and Type 0. When the model misclassifies sequences generated by Type 2 pairs, it will predict that these sequences were generated by Type 0 pairs.
- **Condition 2** If patterns *do not exist* between sequences preceding conflict, the empirical model will struggle to differentiate between Type 2 and Type 1 pairs. When the model misclassifies sequences generated by Type 2 pairs, it will predict that these sequences were generated by Type 1 pairs.

**Fig 1:**
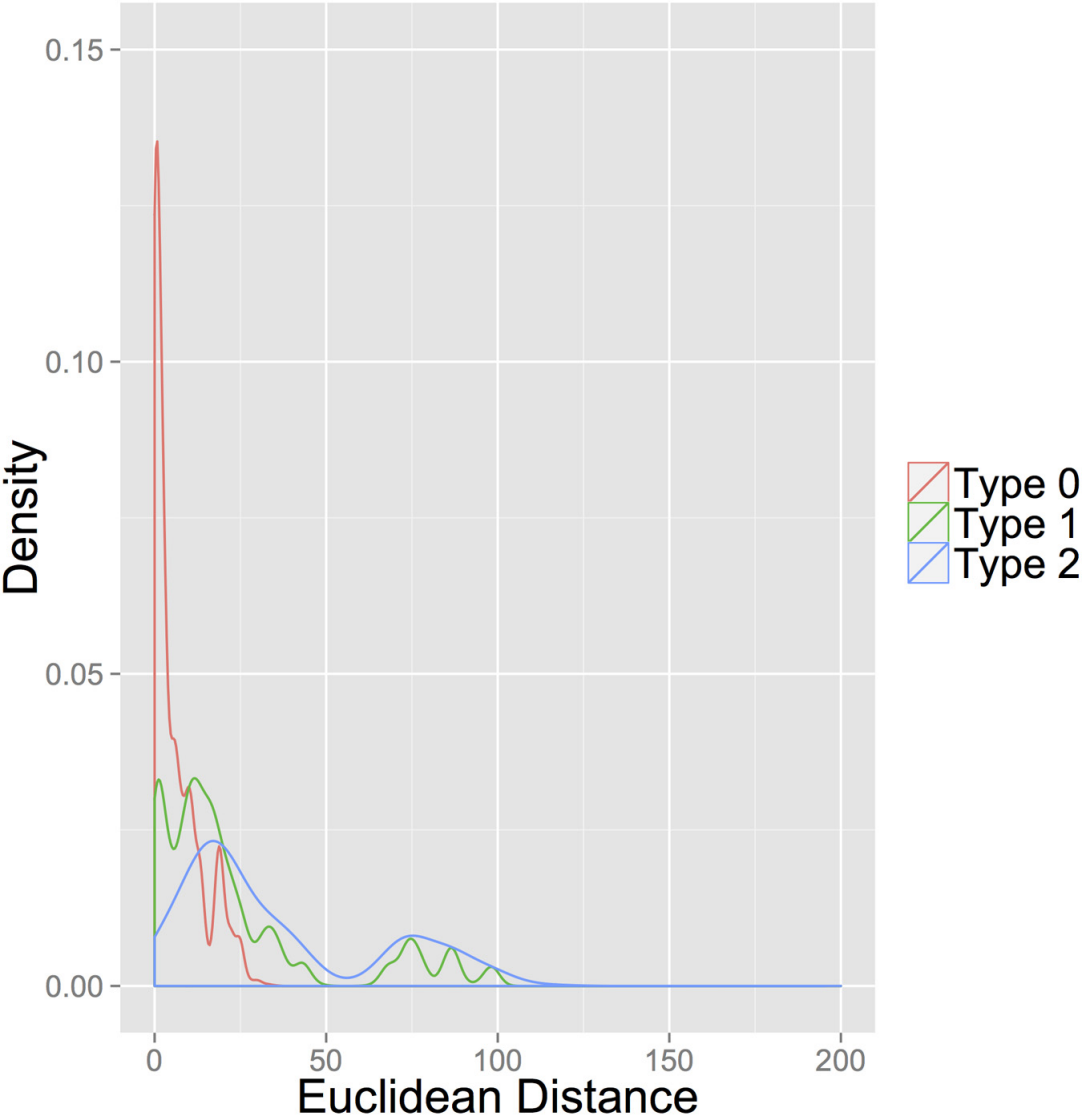
Weekly Euclidean Distances.

**Fig 2:**
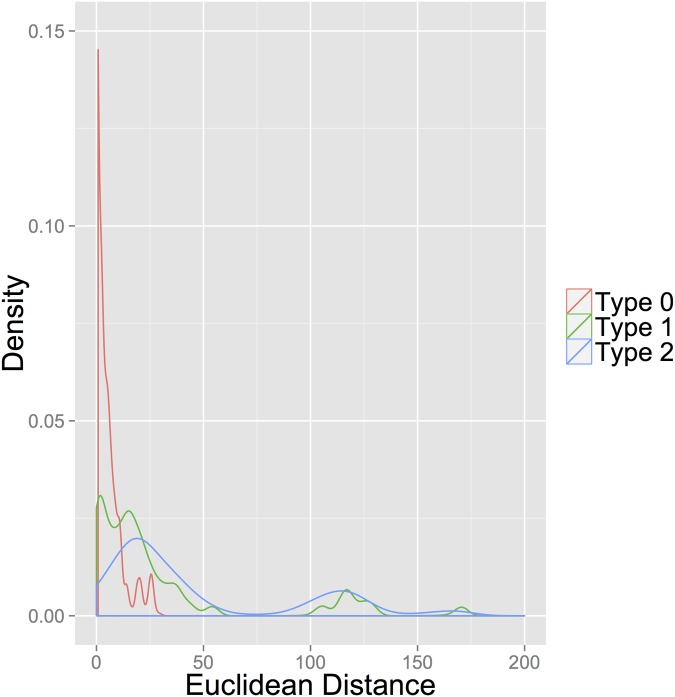
Monthly Euclidean Distances.

The histograms in Figs 1 and 2 reflect the distribution of distances by pair type for the weekly and monthly aggregations using the Euclidean distance. Since the distributions of the eight distances are very similar, they are summed to add mass to the histograms to better illustrate the differences among pair types. The correlation of various action categories is a common feature of event data research and has been reported in a number of studies [16, 27, 52].

These histograms suggest two important findings. First, distances generated by Type 0 pairs tend to be small and are clustered around 0, supporting our assumption that sequences that precede peace are similar. Second, if strong patterns existed between sequences preceding intra-state conflict onsets, then we would expect to see a spike in distribution density of the Type 2 histogram near 0. Clearly, no such spike exists. Instead, these histograms illustrate that distances generated by Type 2 pairs appear more similar to those generated by Type 1 pairs than by Type 0 pairs. Although Figs 1 and 2 provide strong visual evidence suggesting support for Condition 2, we next present an empirical model to test for which condition is being met.

### Regression and Classification Analysis

To more rigorously test for similarities between pair types, we estimate a bivariate logistic regression using our distance measures as regressors and include a control for the number of ongoing crises. The bivariate logistic regression is an appropriate choice because there are two outcome variables for each pair: conflict onset in State A and conflict onset in State B [53]. The bivariate logit allows us to not only calculate the marginal probabilities of *Y*_*A*_ and *Y*_*B*_ (i.e., the probability of onset in State *A* and the probability of onset in State *B* independent of one another), but it also estimates the joint probabilities of *Y*_*A*, *B*_ (i.e., the probability of (no) conflict in both states), providing us with an estimate of the relationship between the two marginal probabilities of onset. Furthermore, since the regressors reflect the distance between two sequences, each of which has its own outcome, we expect that the marginal probabilities of *Y*_*A*_ and *Y*_*B*_ are strongly correlated, making the bivariate logit a theoretically justified method given our data.

$$\begin{array}{ccc} Y_{A,B} & = & \beta_{0.1}+\beta_{0.2}+\beta_{1}VerbCoop_{GOV-REB}+\beta_{2}VerbConfl_{GOV-REB}+\beta_{3}MaterCoop_{GOV-REB}\\ & & +\beta_{4}MaterConfl_{GOV-REB}+\beta_{5}VerbCoop_{REB-OTH}+\beta_{6}VerbConfl_{REB-OTH}\\ & & +\beta_{7}MaterCoop_{REB-OTH}+\beta_{8}MaterConfl_{REB-OTH}+\beta_{9}NumCrisis+\epsilon \end{array}$$ (3)

We use the bivariate logit to classify pair types according to the following three steps. First, we estimate our model on training data consisting of a random sample of half our observations. The regressors are the eight distance measures and a control variable to account for the number of ongoing conflicts during each state-month. The specification can be seen in Eq (3), and all estimates are computed using the VGAM package in R version 2.14.2. Second, using the estimates from the training data, predicted probabilities are calculated for each pair type using the remaining data. However, the bivariate logit provides four predicted probabilities because we have two dichotomous dependent variables—*Y*_*A*_ ∈ {0,1} and *Y*_*B*_ ∈ {0,1}—and therefore four potential outcomes: *Y*_*A*, *B*_ ∈ {(0,0);(0,1);(1,0);(1,1)}. The predicted probabilities for the Type 0 and Type 2 pairs are the predicted probabilities *Y*_*A*, *B*_ = (0,0) and *Y*_*A*, *B*_ = (2,2), respectively. For Type 1 pairs, the predicted probability is equal to the sum of the predicted probabilities of *Y*_*A*, *B*_ = (1,0) and *Y*_*AB*_ = (0,1) because the distance from State A to State B is the same as the distance from State B to State A (non-directional). Finally, our decision rule is to classify each pair by the highest predicted probability. For example, if the predicted probabilities of a Type 0, Type 1, and Type 2 pair are 0.7, 0.2, and 0.1 respectively, the rule classifies Type 0 to be positive while Type 1 and Type 2 are negative. The decision rule is formalized in Eq (4).

$$\begin{array}{c} \hat{PT}=\{\begin{array}{ccccc} 0 & if & pr(Y_{0,0}) & > & pr\left(Y_{0,1}\right)+pr\left(Y_{1,0}\right)\;\text{and}\;pr\left(Y_{1,1}\right)\\ 1 & if & pr\left(Y_{0,1}\right)+pr\left(Y_{1,0}\right) & > & pr\left(Y_{0,0}\right)\;\text{and}\;pr\left(Y_{1,1}\right)\\ 2 & if & pr(Y_{1,1}) & > & pr\left(Y_{0,0}\right)\;\text{and}\;pr\left(Y_{0,1}\right)+pr\left(Y_{1,0}\right) \end{array} \end{array}$$ (4)

The estimates from the regression model are not particularly relevant for our purposes because we are concerned with the model’s ability to distinguish among the three pair types and are not interested in the effect or explanatory power of any variable or set of variables. Additionally, high collinearity exists between the underlying count variables, which further complicates the interpretation of the coefficients. Therefore, we focus on our model’s out-of-sample classification accuracy rather than the specific covariates and their empirical significance.

The predicted and true classes for the out-of-sample observations are shown in a confusion matrix in Table 4 for the Euclidean distance. From these statistics, performance measures detailing the model’s ability to classify out-of-sample observations are calculated and reported in Table 5. Taken together, the confusion matrix and the performance measures enable the analysis of which pair types are distinguishable based on event sequences, which allows us to determine whether Condition 1 or Condition 2 receives empirical support. The performance measures of sensitivity, specificity, positive predictive value (PPV) and negative predictive value (NPV) are used to assess our out-of-sample classification accuracy:

- Sensitivity—the percent of all positive observations correctly classified
  $$\begin{array}{c} \frac{\#ofTruePositives}{\#ofTruePositives+\#ofFalseNegatives} \end{array}$$
- Specificity—the percent of all negative observations correctly classified
  $$\begin{array}{c} \frac{\#ofTrueNegatives}{\#ofFalsePositives+\#ofTrueNegatives} \end{array}$$
- Positive Predictive Value—the percent of positive predictions that are accurate
  $$\begin{array}{c} \frac{\#ofTruePositives}{\#ofTruePositives+\#ofFalsePositives} \end{array}$$
- Negative Predictive Value—the percent of negative predictions that are accurate
  $$\begin{array}{c} \frac{\#ofTrueNegatives}{\#ofFalseNegatives+\#TrueNegatives} \end{array}$$

**Table 4:** Confusion Matrix of Bivariate Logistic Classification.

*Monthly Euclidean*		**Predicted Class**			
		Type 0	Type 1	Type 2	Total
**True Class**	Type 0	3034	4	0	3038
	Type 1	1091	1500	353	2944
	Type 2	85	289	325	699
	Total	4210	1793	678	6681
*Weekly Euclidean*		**Predicted Class**			
		Type 0	Type 1	Type 2	Total
**True Class**	Type 0	3183	0	0	3183
	Type 1	1151	1524	358	3033
	Type 2	93	303	316	712
	Total	4427	1827	674	6928

**Table 5:** Performance of Bivariate Logistic Classification.

	**Performance Measure**	**Type 0**	**Type 1**	**Type 2**
**Monthly**	Sensitivity	99.87	50.95	46.49
	Specificity	50.10	89.88	75.79
	Pos. Pred. Value	72.07	83.66	47.94
	Neg. Pred. Value	99.84	70.46	93.77
**Weekly**	Sensitivity	100	50.25	44.38
	Specificity	49.13	89.83	75.72
	Pos. Pred. Value	71.90	83.42	46.88
	Neg. Pred. Value	100	70.42	93.67

Test-N = 6,681 (Monthly) and 6,928 (Weekly); values are percentages.

For our purposes, we care about the model’s ability to distinguish among types. Therefore, we would like to see that our model actually classifies observations into all three pair types. As can be seen in Table 4, at the weekly level 4,427 are predicted to be Type 0, 1,827 to be Type 1, and 674 to be Type 2, which places enough predictions in each class to move forward.

#### Classification Results for Euclidean Distances

Tables 4 and 5 provides a number of important results. First, we can distinguish between *most* pairs of sequences preceding peace and *most* sequences preceding conflict, but not all. As apparent by our the near-perfect sensitivity, the model over-predicts Type 0 pairs. Recall that for Type 0 pairs, the distances tend to be small, indicating that periods preceding peace are characterized primarily by the absence of events involving domestic rebel groups. Given this, the model is classifying most or all observations with similarly small distances as having been generated by Type 0 pairs. These “masked” conflicts are discussed in greater detail below.

The sensitivity for Type 1 and Type 2 pairs is relatively low, at 50% and 44%, respectively. This means our model classifies just over half of all Type 1 pairs correctly and just under half of all Type 2 pairs correctly. However, the PPV for Type 1, approximately 83%, is large. This is in comparison to 72% for Type 0 and about 48% for Type 2. Overall, this suggests that while we misclassify a large proportion of Type 1 pairs, when the model classifies a pair as Type 1 it is correct 83% of the time. Although our model performs well classifying Type 0 pairs, it struggles to accurately classify Type 2 pairs. When it predicts a pair to be of Type 2, it is correct less than half of the time.

Together, these measures indicate that our model struggles to correctly predict when an observation is a Type 2 pair. The question of central importance to this paper is whether Type 2 pairs are misclassified as Type 0 (i.e., Condition 1) or as Type 1 (i.e., Condition 2). In total, our dataset contains 712 Type 2 pairs using weekly level sequences, of which we misclassify 396. Among these, our model predicts 303 to be Type 1 and only 93 to be Type 0, which is a ratio of greater than 3:1. This provides strong support for Condition 1 and suggests that, based on our data and research design, we are unable to identify common patterns between event sequences that precede onsets of intra-state conflict.

Our findings on the patterns of escalation can be thought of using a medical analogy. Consider a random sample of patients whose vital signs are monitored weekly. Variation between the vital signs of healthy patients (i.e., Type 0 pairs) is likely to be low; all will have a temperature of about 98.6, they will not feel stomach or chest paints, etc. However, the distance between the vital signs of a patient who continues to maintain health and a patient who is falling ill is likely to be large. Additionally, the distance between vital sign sequences of two patients approaching an illness (i.e., a Type 2 pair) may be quite high, as different illnesses may alter vital signs in very different ways.

#### Classification Results for Levenshtein and Mutual Information

Tables 6 and 7 detail the classification of the bivariate logit model when sequence similarity is scored using the Levenshtein distance and mutual information, both at the weekly level of aggregation. In general, the results are quite robust to the metric used and the primary finding, that Type 2 pairs continue to be classified as Type 1, remains strong in both experiments. In fact, these metrics *strengthen* the finding. When using the Levenshtein distance, there are 3.76 times more Type 2 pairs classified as Type 1 than there are Type 2 pairs classified as Type 0. When using the mutual information metric, there are 7.35 times more Type 2 pairs predicted to be Type 1 than there are Type 2 pairs predicted to be Type 0. In the Euclidean model, this number is 3.26. Thus, both metrics, and especially mutual information, provides *more* support for Condition 2 (that Type 2 pairs appear more like Type 1 than Type 0) than the Euclidean metric.

**Table 6:** Confusion Matrix Using Levenshtein and Mutual Information.

*Weekly Levenshtein*		**Predicted Class**			
		Type 0	Type 1	Type 2	Total
**True Class**	Type 0	3080	103	0	3183
	Type 1	1137	1652	244	3033
	Type 2	92	346	274	712
	Total	4309	2101	518	6928
*Weekly Mutual Info*.		**Predicted Class**			
		Type 0	Type 1	Type 2	Total
**True Class**	Type 0	2953	230	0	3183
	Type 1	855	1909	269	3033
	Type 2	49	360	303	712
	Total	3857	2499	572	6928

**Table 7:** Performance Using Levenshtein and Mutual Information.

	**Performance Measure**	**Type 0**	**Type 1**	**Type 2**
**Levenshtein**	Sensitivity	96.76	54.47	38.48
	Specificity	51.43	86.11	76.13
	Pos. Pred. Value	71.48	78.63	52.90
	Neg. Pred. Value	96.07	71.39	93.17
**Mutual Information**	Sensitivity	92.77	62.94	42.56
	Specificity	59.07	83.59	78.22
	Pos. Pred. Value	76.56	76.39	52.97
	Neg. Pred. Value	92.51	74.62	93.57

Test-N = 6,928 weekly observations; values are percentages.

Scoring the sequences using mutual information provides an additional interesting finding that should be explored in future research. Specifically, relative to the Euclidean and Levenshtein models, the mutual information model is considerably more likely to predict Type 1 pairs. Of the 6,928 out-of-sample observations, 2,499 are predicted to be Type 1 whereas just 1,827 are predicted to be Type 1 using the Euclidean metric. While the number of correctly identified Type 1 pairs is greatest for mutual information, its PPV is just 76%, compared with 79% for Levenshtein and 83% for Euclidean. Furthermore, 230 predicted Type 1 pairs are in truth Type 0, where the Levenshtein model mispredicts 103 such cases, and the Euclidean model mispredicts 0 of them. This suggests that the mutual information metric captures a relationship not represented, or represented less, by the other metrics. In future work, perhaps combinations of these classes of metrics—geometric distances, edit distances, and entropy-based—could be exploited in an ensemble method that leverages the strengths of each.

#### ‘Masked’ Conflict

Although each model predicts the majority of misclassified Type 2 pairs to be Type 1, they still predict 93 (Euclidean), 92 (Levenshtein), and 49 (mutual information) to be of Type 0. In the Euclidean model, of the 3,033 Type 1 pairs, 1,151 are misclassified to be Type 0. However, at the weekly level Type 0 pairs are *never* misclassified as Type 1 *or* Type 2. The intuition is that these misclassified conflict-preceding sequences are quantitatively similar to peace-preceding sequences. In other words, they are characterized predominantly by the absence of events involving rebels. These “masked” conflicts present a challenge for event data forecasting models.

We suggest two factors that may be responsible for masked conflicts. First, it is possible that some conflict onsets are not preceded by politically relevant events and occur spontaneously. For example, we believe the Tunisian Revolution that began in December, 2010 would fall into this class of conflict. However, despite the lack of observable “events”, it is possibly that changes in popular sentiments occurred prior to the onset of conflict. Moving forward, it may be possibly to capture variation in sentiments that may occur in the absence of actual events through content analysis of social media outlets like Facebook and Twitter.

Second, rebel activity may be occurring but not reported in the news. This is possible in places where rebel activity is so common that it becomes no longer newsworthy, as may be the case in the Philippines in the early 2000s. Additionally, non-reporting of actual events may occur in regions with minimal or non-existent formal journalistic reporting. ICEWS’ use of 75 international news sources is an attempt to ameliorate this concern; regardless, there will always be reporting bias in published sources. For instance, it is highly unlikely for a Hezbollah attack in Tel Aviv to go unreported, but much easier for violence on the Solomon Islands to go unreported. When modeling events, zero-inflated models [54] or occupancy models [55] could be used to correct for the disproportionate share of zeros. However, we are referring to the more general concern of sparse data introducing a form of measurement error into the event data. Although it is difficult to account for a lack of reporting, the mutual information metric would account for lower levels of reporting in such places, and this may be one reason why that model predicts just 49 Type 2 pairs to be Type 0 while the Euclidean and Levenshtein predict 93 and 92, respectively.

**Fig 3:**
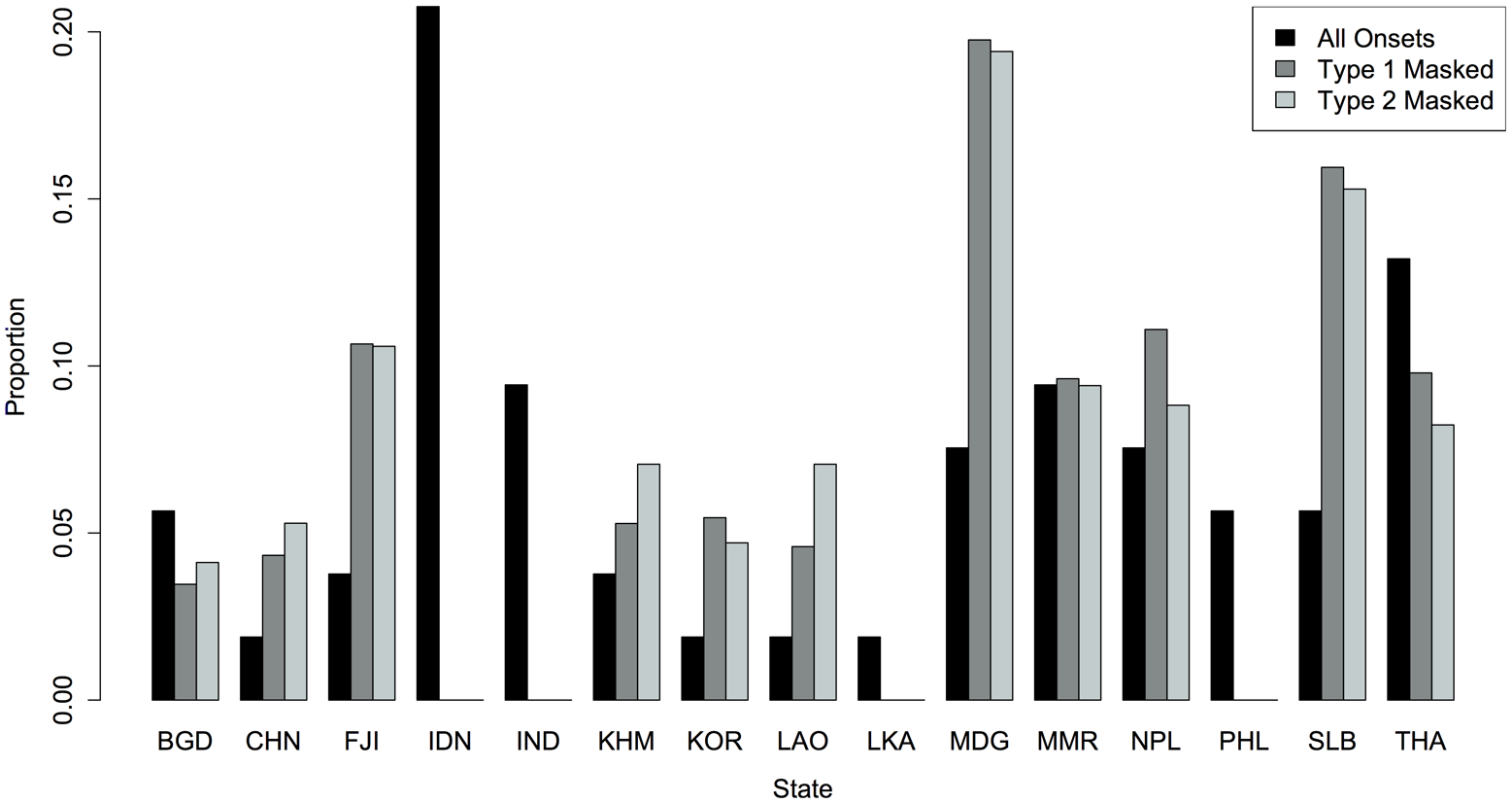
Proportion of Onsets and Masked Conflicts by State.

To illustrate where masked conflicts occur, Fig 3 is a side-by-side-by-side bar plot by state using the Euclidean model. The black bar corresponds to the state’s proportion of the 53 conflict onsets. The dark gray bar, or the middle bar, reflects to the state’s proportion of onset sequences in Type 1 pairs that have been misclassified as Type 0. The light gray bar indicates the state’s proportion of onset sequences in Type 2 pairs that have been misclassified as Type 0. As expected, the dark gray and light gray bars are almost identical.

If the masked conflicts were dispersed randomly by state, the black and gray bars would be roughly the same for all states. However, we see that several states have a disproportionately high share of masked conflicts while others have a disproportionately low share. The states that appear most associated with masked conflicts are Fiji [FIJ], Madagascar [MDG], and the Solomon Islands [SLB]. This is seen by the disproportionately high gray bars in comparison to the black bar for each state. As suggested above, these states are associated with masked conflicts primarily because of the absence of reporting, and thus present a challenge for event data studies. On the other hand, masked conflicts do not occur in Indonesia [IDN], India [IND], Sri Lanka [LKA] or the Philippines [PHL], which the absence of both dark and light gray bars for these states demonstrates. The absence of masked conflicts in these states, which likely receive more comprehensive reporting, suggests potential media bias.

## Conclusion

The central goal of this study is to rigorously analyze the extent to which similarities exist between sequences of events that precede the onset of intra-state conflict in various states over time. To achieve this, we introduce an application of sequence analysis that allows us to leverage the strengths of both large-N and event data studies in order to most effectively address our central question. Using the ICEWS data, we construct and compare sequences of event counts based on the activities of government and opposition forces. The results lead to two important findings.

First, our models over-predict Type 0, misclassifying both Type 1 and Type 2 pairs. That is, pairs of sequences where one or both precede conflict appear similar to pairs where both precede peace. We hypothesize that this occurs due to “masked” conflicts, which occur either spontaneously and are not preceded by prior political events, or are preceded by relevant events that are simply not reported. Moving forward, we believe that content posted in social media outlets such as Facebook and Twitter may provide information regarding shifts in popular sentiments that may occur in the absence of actual events. Thus, accounting for sentiments may allow researchers greater leverage to predict “spontaneous” masked conflicts, which they would otherwise be unable to do relying solely on event data.

Second, and of central importance to this paper, is that the bivariate logit models tend to incorrectly predict Type 2 pairs to be Type 1. This indicates that sequences preceding peace exhibit identifiable patterns and may be related to the fact that peace is best characterized by the absence of conflict, while conflict takes many forms and exhibits a variety of different event structures. This is an important contribution to both academics and policy analysts who are interested in intra-state conflict because it suggests that although certain structural variables may have consistent effects of conflict onsets across time and space, *the actual events that comprise the escalation towards conflict may be unique to the conflict*.

For the academic community, this raises important questions, such as what are the causal mechanisms connecting structural conditions like regime type or GDP to actual day-to-day events. For policy analysts, this finding may suggest that less weight should be given to identifying historical similarities when attempting to forecast conflict onset. Like in most quantitative studies of conflict, this finding is contingent on the accuracy of our data, aggregation strategies, and choice of empirical model. Cognizant of this, we include numerous robustness checks, including performing all empirical analyses on both weekly and monthly levels of temporal aggregation, which all support our central findings. However, given the long qualitative literature suggesting that similarities between historical cases do exist (see [56, 57] and [58]), we do not want to dismiss the possibility that generalizable patterns of events may exist in a dataset with greater temporal and spatial coverage. Overall, we hope that this study demonstrates that objective comparisons of nuanced event sequences are able to generate substantively important findings and encourages future analysis of the actual events that precede onsets of conflict.

## Supporting Information

S1 Table Replication with UCDP Data. Replication of the analysis using the Uppsala Conflict Data Project’s Armed Conflict dataset [59].
(PDF)

## References

[1] Moore WH. Repression and Dissent: Substitution, Context, and Timing. American Journal of Political Science. 1998;42(3):851–873. 10.2307/2991732

[2] Schrodt PA. Parallel Event Sequences in International Relations. Political Behavior. 1990;12(2):97–123. 10.1007/BF00992465

[3] Moore WH, Lindstrom R. The Violent Intranational Conflict Data Project (VICDP) Codebook; 1996 University of California Riverside, typescript.

[4] Shellman S. Process Matters: Conflict and Cooperation in Sequential Government-Dissident Interactions. Security Studies. 2007;15(4):563–599. 10.1080/09636410601184603

[5] Schrodt PA. Pattern Recognition of International Event Sequences: A Machine Learning Approach In: Hudson V, editor. Artificial Intelligence and International Politics. Boulder: Westview; 1991.

[6] Schrodt PA, Gerner DJ. Analyzing International Event Data: A Handbook of Computer-Based Techniques; 2012. *http://goo.gl/yZGg8S*.

[7] Collier P, Hoeffler A. Greed and Grievance in Civil War. Oxford Economic Papers. 2004;56:563–595. 10.1093/oep/gpf064

[8] Fearon JD, Laitin DD. Ethnicity, Insurgency, and Civil War. American Political Science Review. 2003;97(1):75–90. 10.1017/S0003055403000534

[9] Shearer R. Forecasting Israeli-Palestinian Conflict with Hidden Markov Models; 2006. *http://eventdata.parusanalytics.com/papers.dir/Shearer.IP.pdf*.

[10] Schrodt PA, Gerner DJ. Cluster-Based Early Warning Indicators for Political Change in the Contemporary Levant. American Political Science Review. 2000;94(4):803–817. 10.2307/2586209

[11] Pevehouse JC, Goldstein JS. Serbian Compliance or Defiance in Kosovo? Statistical Analysis and Real-Time Predictions. The Journal of Conflict Resolution. 1999;43(4):538–546. 10.1177/0022002799043004007

[12] Schrodt PA. Forecasting Conflict in the Balkans using Hidden Markov Models In: Trappl R, editor. Programming for Peace: Computer-Aided Methods for International Conflict Resolution and Prevention. Dordrecht, Netherlands: Kluwer Academic Publishers; 2006 p. 161–184.

[13] Hammerli A, Gattiker R, Weyermann R. Conflict and Cooperation in an Actor’s Network of Chechnya based on Event Data. Journal of Conflict Resolution. 2006;50(159):159–175. 10.1177/0022002705284826

[14] O’Brien S. Crisis Early Warning and Decision Support: Contemporary Approaches and Thoughts on Future Research. International Studies Review. 2010;12(1):87–104. 10.1111/j.1468-2486.2009.00914.x

[15] Schrodt PA. Automated Production of High-Volume, Near-Real-Time Political Event Data; 2010. Presented at the American Political Science Association meetings, Washington.

[16] Gerner DJ, Schrodt PA, Francisco RA, Weddle JL. The Machine Coding of Events from Regional and International Sources. International Studies Quarterly. 1994;38:91–119. 10.2307/2600873

[17] Schrodt PA, Gerner DJ. Validity Assessment of a Machine-Coded Event Data Set for the Middle East, 1982–1992. American Journal of Political Science. 1994;38:825–854. 10.2307/2111609

[18] Schrodt PA. TABARI: Textual Analysis By Augmented Replacement Instructions; 2014. *http://goo.gl/72XFle*.

[19] King G, Lowe W. An Automated Information Extraction Tool for International Conflict Data With Performance as Good as Human Coders: A Rare Events Evaluation Design. International Organization. 2003;57(3):617–642. 10.1017/S0020818303573064

[20] King G. 10 Million International Dyadic Events; 2003. *http://hdl.handle.net/1902.1/FYXLAWZRIA*.

[21] Van Brackle D, Haglich P. Improvements in the Jabari event coder. Advances in Design for Cross-Cultural Activities. 2012;13:191.

[22] Schrodt PA, Gerner DJ, Yilmaz O. Conflict and Mediation Event Observations (CAMEO): An Event Data Framework for a Post Cold War World In: Bercovitch J, Gartner S, editors. International Conflict Mediation: New Approaches and Findings. New York: Routledge; 2009.

[23] Bond D, Jenkins JC, Taylor CL, Schock K. Mapping Mass Political Conflict and Civil Society: Issues and Prospects for the Automated Development of Event Data. Journal of Conflict Resolution. 1997;41(4):553–579. 10.1177/0022002797041004004

[24] Harff B, Gurr TR. Systematic Early Warning of Humanitarian Emergencies. Journal of Peace Research. 2001;35(5):359–371.

[25] Davies JL, Harff B, Speca AL. Dynamic Data for Conflict Early Warning In: Davies JL, Gurr TR, editors. Preventive Measures: Building Risk Assessment and Crisis Early Warning. Lanham, MD: Rowman and Littlefield; 1998 p. 79–94.

[26] Jenkins CJ, Bond D. Conflict Carrying Capacity, Political Crisis, and Reconstruction. Journal of Conflict Resolution. 2001;45(1):3–31. 10.1177/0022002701045001001

[27] Goldstein JS. A Conflict-Cooperation Scale for WEIS Events Data. Journal of Conflict Resolution. 1992;36:369–385. 10.1177/0022002792036002007

[28] Schrodt PA. Inductive Event Data Scaling using Item Response Theory; 2007. Presented at the Summer Meeting of the Society of Political Methodology.

[29] Schrodt PA, Gerner DJ, Abu-Jabr R, Yilmaz O, Simpson EM. Analyzing the Dynamics of International Mediation processes in the Middle East and Balkans; 2001. Presented at the American Political Science Association meetings, San Francisco.

[30] Schrodt PA, Gerner DJ. An Event Data Analysis of Third-Party Mediation. Journal of Conflict Resolution. 2004;48(3):310–330. 10.1177/0022002704264137

[31] Duval RD, Thompson WR. Reconsidering the Aggregate Relationship between Size, Economic Development, and Some Types of Foreign Policy Behavior. American Journal of Political Science. 1980;24(3):511–525. 10.2307/2110830

[32] Brandt PT, Freeman JR. Advances in Baysian time Series Modeling and the Study of Politics: Theory testing, Forecasting, and Policy Analysis. Political Analysis. 2005;14:1–36. 10.1093/pan/mpi035

[33] Shellman S, Stewart B. Political Persecution or Economic Deprivation? A Time-Series Analysis of Haitian Exodus, 1990–2004. Conflict Management and Peace Science. 2007;24:121–137. 10.1080/07388940701257523

[34] Ward MD, Greenhill BD, Bakke KM. The Perils of Policy by P-Value: Predicting Civil Conflicts. Journal of Peace Research. 2010;47(5).

[35] Alt J, King G, Signorino CS. Aggregation among Binary, Count, and Duration Models: Estimating the Same Quantities from Different Levels of Data. Political Analysis. 2001;9:21–44. 10.1093/oxfordjournals.pan.a004863

[36] Thomas DG. Event Data Analysis and Threats from Temporal Aggregation; 2002. Presented at the Florida Political Science Association Meeting, Sarasota.

[37] Shellman S. Time Series Intervals and Statistical Inference: The Effects of Temporal Aggregation on Event Data Analysis. Political Analysis. 2004;12(1):97–104. 10.1093/pan/mpg017

[38] Kovar K, Petrak J, Pfahringer B, Trappl R, Widmer G. Searching for Patterns in Political event Sequences: Experiments with the KEDS Database. Cybernetics and Systems. 2000;31(6).

[39] Lustick IS, Alcorn B, Garces M, Ruvinsky A. From Theory to Simulation: The Dynamic Political Hierarchy in Country Virtualization Models; 2010. Presented at the Annual Meeting of the American Political Science Association.

[40] Montgomery JM, Hollenbach FM, Ward MD. Improving Predictions Using Ensemble Bayesian Model Averaging. Political Analysis. 2012;20(3):271–291. 10.1093/pan/mps002

[41] Schrodt PA. Forecasting Political Conflict in Asia Using Latent Dirichlet Allocation Models; 2011. Presented at the Annual Meeting of the European Political Science Association.

[42] Signorino CS, Ritter JM. Tau-b or Not Tau-b: Measuring the Similarity of Foreign Policy Positions. International Studies Quarterly. 1999;43(1):115–144. 10.1111/0020-8833.00113

[43] Villaverde AF, Ross J, Banga JR. Reverse Engineering Cellular Networks With Information Theoretic Methods. Cells. 2013;2(2):306–329. 10.3390/cells2020306 24709703 PMC3972682

[44] Reshef DN, Reshef YA, Finucane HK, Grossman SR, McVean G, Turnbaugh PJ, et al Detecting Novel Associations in Large Data Sets. Science. 2011;334(6062):1518–1524. 10.1126/science.1205438 22174245 PMC3325791

[45] Drozdova K, Samoilov M. Predictive Analysis of Concealed Social Network Activities Based on Communication Technology Choices: Early-Warning Detection of Attack Signals From Terrorist Organizations. Computational and Mathematical Organization Theory. 2010;16(1):61–88. 10.1007/s10588-009-9058-2

[46] Hodge VJ, Austin J. A Comparison of Standard Spell Checking Algorithms and a Novel Binary Neural Approach. IEEE Transaction on Knowledge and Data Engineering. 2003;15(5):1073–1081. 10.1109/TKDE.2003.1232265

[47] van Noord G, Gerdemann D. An Extendible Regular Expression Compiler for Finite-State Approaches in Natural Language Processing In: Boldt O, Jorgensen H, editors. Automata Implementation. vol. 2214 of Lecture Notes in Computer Science. Springer Berlin / Heidelberg; 2001 p. 122–139.

[48] Troncoso-Pastoriza JR, Katzenbeisser S, Celik M. Privacy Preserving Error Resilient DNA Searching Through Oblivious Automata. In: Proceedings of the 14th ACM conference on Computer and communications security. New York, NY: ACM; 2007. p. 519–528.

[49] Saha S, Bridges S, Magbanua Z, Peterson D. Computational Approaches and Tools Used in Identification of Dispersed Repetitive DNA Sequences. Tropical Plant Biology. 2008;1:85–96. 10.1007/s12042-007-9007-5

[50] Cover TM, Thomas JA. Elements of information theory. John Wiley & Sons; 2012.

[51] Linfoot E. An informational measure of correlation. Information and control. 1957;1(1):85–89. 10.1016/S0019-9958(57)90116-X

[52] Schrodt PA, Gerner DJ. Empirical Indicators of Crisis Phase in the Middle East, 1979–1995. Journal of Conflict Resolution. 1997;25(4):803–817.

[53] McCullagh P, Nedler JA. Generalized Linear Models, Second Edition. New York: Chapman and Hall; 1989.

[54] Bagozzi, BE. Forecasting Civil Conflict with Zero-Inflated Count Models; 2011. *http://www.benjaminbagozzi.com/research.html*.

[55] MacKenzie DI, Nichols JD, Hines JE, Knutson MG, Franklin AB. Estimating Site Occupancy, Colonization, and Local Extinction when a Species is Detected Imperfectly. Ecology. 2003;84(8):2200–2207. 10.1890/02-3090

[56] May ER. “Lessons” of the Past: The Use and Misuse of History in American Foreign Policy. New York: Oxford University Press; 1973.

[57] Khong YF. Analogies at War. Princeton: Princeton University Press; 1992.

[58] Neustadt RE, May ER. Thinking in Time: The Uses of History for Decision Makers. New York: Free Press; 1986.

[59] Themnér L, Wallensteen P. Armed conflicts, 1946–2013. Journal of Peace Research. 2014;51(4):541–554. 10.1177/0022343314542076

