# Supplementary material for: Kickoff to Conflict: A Sequence Analysis of Intra-State Conflict-Preceding Event Structures
Source: PLoS One. 2015 May 7;10(5):e0122472. doi: 10.1371/journal.pone.0122472 (PMC4424002; doi:10.1371/journal.pone.0122472)
Supplement: S1 Table — Replication of the analysis using the Uppsala Conflict Data Project’s Armed Conflict dataset [59]. (PDF) [file pone.0122472.s001.pdf]

Supporting Information

- **S1 Table: Replication with UCDP Data.**

The analysis has been replicated using the Uppsala Conflict Data Project's Armed Conflict dataset [59]. These data measure episodes of intra-state conflict at the yearly level, where a conflict onset is identified at the date when the 25 battle-death threshold has been passed. The episode of conflict is considered ongoing until the end date or, when no end date is reported, the end of the calendar year.

Due to the limited scope of the ICEWS event data, there are just 19 identified episodes of conflict onset in UCDP, yielding just 105 Type 2 pairs, 540 Type 1 pairs, and 630 Type 0 pairs. Despite the limited number of observations, some of the results are quite consistent with the GTDS data, particularly for Type 0 predictions. With respect to the UCDP data, of those predicted to be Type 0, 59% are in truth Type 0, 36% are Type 1, and 5% are Type 2. For the GTDS data, of those predicted to be Type 0, 72% are 0, 26% are Type 1, and 2% are Type 2. Given the low number of observations in the UCDP data, we do not believe these trends to be inconsistent.

For Type 1 predictions, the trend is somewhat inconsistent, particularly because there are 97 cases where the model predicts Type 1 when the pair is actually a Type 0. This means these pair distances appear as if one is experiencing conflict when there is no conflict. This *never* occurs with the GTDS Euclidean model, but does for the Levenshtein and mutual information models. One explanation for this difference is that the UCDP data are measured at a coarser level of granularity than the GTDS. The event data sequences, then, may be accurately observing low levels of conflict that would be captured in the GTDS but are not captured in the UCDP.

As has been found in the primary experiments, Type 2 predictions are relatively rare. This, coupled with the greatly reduced number of Type 2 observations, leads to just one Type 2 prediction. Although it is correct, there are simply not enough Type 2 predictions to make any meaningful inferences. With respect to the misclassification of pairs that are in truth Type 2, using the UCDP data we find that 35 are predicted to be Type 1 and 18 are predicted to be Type 0. This ratio of about 2:1 is smaller than the roughly 3:1 reported by the Euclidean model, and considerably smaller than the 7:1 reported by the mutual information model. However, Type 2 pairs still appear more similar to Type 1 than Type 0, and thus the UCDP robustness check still supports Condition 2, despite the lack of observations.

S1 Table: Replication with UCDP Data

Confusion Matrix Using UCDP Data

Weekly Euclidean		*Predicted Class*			
		Type 0	Type 1	Type 2	Total
True Class	Type 0	224	97	0	321
	Type 1	137	111	0	248
	Type 2	18	35	1	54
	Total	379	243	1	623

Performance with UCDP Data

	Performance Measure	*Type 0*	*Type 1*	*Type 2*
Weekly Euclidean	Sensitivity	69.78	44.76	1.85
	Specificity	37.09	60.00	58.88
	Pos. Pred. Value	59.10	45.68	100
	Neg. Pred. Value	60.25	63.95	91.48

Test-N = 623 (Weekly); values are percentages.
